# Walking adaptability training for individuals after stroke (ATTAINS): study protocol for a randomized, waiting-list controlled trial

**DOI:** 10.1186/s13063-025-09276-w

**Published:** 2025-11-27

**Authors:** Marijne Nieuwelink, Katrijn Smulders, Jip Kamphuis, Jorik Nonnekes, Noël Keijsers, Vivian Weerdesteyn

**Affiliations:** 1 Department of Research, Sint Maartenskliniek, Nijmegen, The Netherlands; 2 Department of Rehabilitation, Donders Institute for Brain, Cognition and Behaviour, Radboud University Medical Center, Nijmegen, The Netherlands; 3 Department of Rehabilitation, Sint Maartenskliniek, Nijmegen, the Netherlands; 4 Department of Sensorimotor Neuroscience, Donders Institute for Brain, Cognition and Behaviour, Radboud University, Nijmegen, The Netherlands

**Keywords:** Walking adaptability, Stroke, Rehabilitation, Treadmill training, Treadmill intervention, Augmented reality

## Abstract

**Background:**

People with stroke (PwS) in the chronic phase often experience difficulties adapting their gait to meet environmental demands. This so-called walking adaptability is important for safe and independent walking in everyday life. Walking adaptability can be trained on a treadmill with augmented reality (C-mill), but conclusive evidence of its effectiveness in PwS is yet lacking. The primary aim of this study is, therefore, to evaluate the efficacy of C-mill-based task-specific training on walking adaptability in a randomized controlled trial. As a secondary aim, we will conduct a responder analysis to explore potential determinants of treatment effects.

**Method:**

This is a single-center, randomized, waiting-list controlled trial. We aim to enroll 84 people in the chronic phase (> 6 months) after a first, unilateral supratentorial stroke, who are able to walk independently for 10 min. Following baseline assessment, participants will be randomly assigned to either the experimental group or the waiting-list control group. The experimental group will receive ten 1-h training sessions on the C-mill targeting walking adaptability in a 5-week period. Participants assigned to the waiting-list controlled group will continue their usual care for 5 weeks; thereafter they will receive the same walking adaptability training as the experimental group. To determine the effects of the training, assessments of walking adaptability, as well as other balance and gait-related tasks, will be conducted at baseline (T0) and following the 5-week intervention/waiting period (T1). To allow responder analysis across both groups, the waiting-list control group will have an additional assessment (T2) following their training period.

**Outcome:**

The two primary outcomes are the time needed to complete the obstacle subtask of the Emory functional ambulation profile (EFAP-obstacle) and the error-corrected time score on the walking adaptability ladder test (WALT). To correct for the two primary outcomes significant effects are assumed for *p* < 0.025. Secondary outcomes include lab-based tests to assess target stepping and gait stability and commonly used clinical tests to assess balance and walking capacity. Furthermore, home-based walking activity and falls are monitored.

**Discussion:**

The results of this study are expected to inform tailored provision of walking adaptability training in PwS in the chronic phase.

**Trial registration:**

ClinicalTrials.gov NCT05827380 (URL: https://clinicaltrials.gov/study/NCT05827380). Final protocol submitted on 18-7-2022, registered on 25-04-2023.

## Introduction

### Background and rationale

After stroke, 80% of survivors regain walking ability [1]. Yet, residual post-stroke gait impairments are highly prevalent, and have a profound impact on activities of daily living and quality of life [2]. Furthermore, gait impairments are a key determinant of frequent falling in people with stroke (PwS), with a majority of falls in the chronic phase after stroke happening during walking activities [3–5]. For safe community ambulation, one needs the ability to adapt gait to meet environmental demands, for example, navigating through a crowded street, or walking on uneven surfaces [6]. Previous studies have demonstrated profound impairments in this so-called walking adaptability after stroke. Yet, despite its evident importance for daily life mobility, there is limited high-grade evidence supporting the effectiveness of task-specific training for improving walking adaptability in PwS.

In community-dwelling older people, a recent meta-analysis of randomized controlled trials showed that walking adaptability training is efficacious in preventing falls in daily life [7]. There is also emerging evidence for its benefits in PwS. A proof-of-concept study in 16 PwS in the chronic phase reported significant improvements on the obstacle subtask of the Emory Functional Ambulation Profile (EFAP), as well as a 25% improvement on a treadmill-based obstacle avoidance task following a 5-week walking adaptability training [8, 9]. Furthermore, two controlled studies have been conducted in PwS that contrasted treadmill-based with overground walking adaptability training [10, 11]. One of these studies found improvements in obstacle avoidance and target stepping post-intervention for both training modalities [10]. The other reported significant training effects on obstacle avoidance following the treadmill-based program only [11]. However, their assessment of walking adaptability (i.e., obstacle avoidance on the treadmill) greatly resembled the tasks used during the treadmill-based training itself. This resemblance limits the ability to draw conclusions about the generalizability of the observed training effects to overground walking contexts. Importantly, neither study included a control group receiving usual care, such that no definitive conclusions could be drawn regarding the efficacy of walking adaptability training in contrast to usual care. While two other randomized controlled trials in PwS did compare walking adaptability training with usual care, these studies did not use a walking adaptability score as an outcome measure [12, 13]. As training effects are to be expected in outcomes that align with the constructs of the actually trained activities [14], including a walking adaptability measure as the primary outcome is imperative for evaluating the efficacy of this type of training. Hence, there is a need for a definitive randomized controlled trial in PwS that (1) contrasts a walking adaptability intervention with usual care and (2) includes a walking adaptability-related primary outcome measure.

The primary objective of this study is to evaluate the efficacy of walking adaptability training using an instrumented treadmill with augmented reality (C-mill) against usual care. The C-mill has been developed to incorporate the principle of task-specific training and to enable a high dose of walking practice per session, which are requirements for effective training [9, 15, 16]. We hypothesize that people receiving the walking adaptability training will improve their walking adaptability when compared to a waiting-list control group receiving usual care. As a secondary objective, we aim to conduct a responder analysis to explore potential determinants (e.g., age, disease severity and balance capacity) of treatment effects. Such insights are expected to inform tailored prescription for increasing the individual’s likelihood of benefitting from walking adaptability training [14].

## Methods

### Regulation statement and ethics approval

The study will be conducted according to the principles of the Declaration of Helsinki (Version 2013) and in accordance with the Medical Research Involving Human Subjects Act (WMO), ICH Guidelines for Good Clinical Practice, and the GDPR. Ethics approval for the study protocol was obtained from the accredited medical ethics committee Arnhem-Nijmegen, the Netherlands (2021–13409, NL80178.091.21). All changes to the protocol will be notified as amendments to the METC that gave a favorable opinion. This study protocol is written according to the SPIRIT guidelines. In addition, the trial is registered on Clinicaltrials.gov (NCT05827380). All items of the WHO Trial Registration Data Set are included in the protocol [17].

### Study design and setting

A single-center, two-armed, superiority, randomized controlled trial will be conducted to determine the efficacy of walking adaptability training using an instrumented treadmill with augmented reality in people in the chronic phase after stroke. The study is conducted at the Sint Maartenskliniek (Ubbergen, the Netherlands).

### Recruitment, selection and consent

Participants will be recruited in several ways. First, individuals who visited the mobility outpatient clinic, or were discharged from inpatient rehabilitation at the Sint Maartenskliniek between 2020 and 2023 will be informed about the study by their treating physician. Second, individuals discharged from the Canisius Wilhelmina Ziekenhuis (CWZ) between 2022 and 2024 will be informed about the study by their treating clinician. Third, members of the regional brain injury network—including rehabilitation physicians, physical therapists, and home care workers—will share brief study information with potentially eligible participants. Fourth, different media platforms—such as newspapers, regional magazines, and social media—will be used to share information about the study with the general public. Further information about the study can be obtained from the research team by phone, email or an online registration form.

Individuals who are interested in participation after having received the detailed study information folder will be further screened regarding inclusion and exclusion criteria through a phone call. Following provisional inclusion, the eligibility criteria will be verified during the intake visit. If all inclusion criteria are met, informed consent is obtained by a researcher of the research team.

### Eligibility criteria

Participants are included if they are in the chronic phase (> 6 months) after a first, unilateral supratentorial stroke, with initial involvement of the lower extremity in the acute phase. Other inclusion criteria are as follows: ability to walk independently for ten or more consecutive minutes (FAC 4–5). Exclusion criteria are as follows: (1) any other neurological or musculoskeletal disorder affecting gait or balance, (2) contractures or spasticity requiring surgery within the duration of the study period, (3) having received multiple training sessions on C-mill or the Gait Real-time Analysis Interactive Lab (GRAIL, Motek Medical BV, the Netherlands) in the 12 months prior to the start of the study, (4) severe cognitive or visuo-spatial impairments limiting comprehension of instructions or correct perception of the walking surfaces and augmented reality environment during assessment and training sessions.

### Group allocation and blinding

Participants will be randomly assigned to either the experimental or the waiting-list control group with a 1:1 allocation ratio. Randomization will be stratified using a Motricity Index lower extremity cut-off score of 64 [18]. The Motricity Index is a clinical assessment to measure the strength of the lower extremity after stroke and will be obtained during the intake assessment [19]. A variable block randomization model (block sizes of 4 and 6) is used to minimize uneven distribution between groups. Randomization is performed in Castor EDC, a web-based data management system for academic studies (www.castoredc.com) to ensure concealed allocation. Randomization is performed by the research coordinator (MN) or an authorized research assistant.

Due to the nature of the intervention, participants cannot be blinded to group allocation. No blinding is necessary for the physical therapists, who carry out the walking adaptability training, because they are not involved in the assessments of the outcome measures. The research coordinator (MN) conducts the assessments, yet the primary outcomes will be scored offline from (muted) video recordings of the assessment by an independent assessor who is blind to group allocation and the timepoint of assessment.

### Participant timeline

**Fig. 1:**
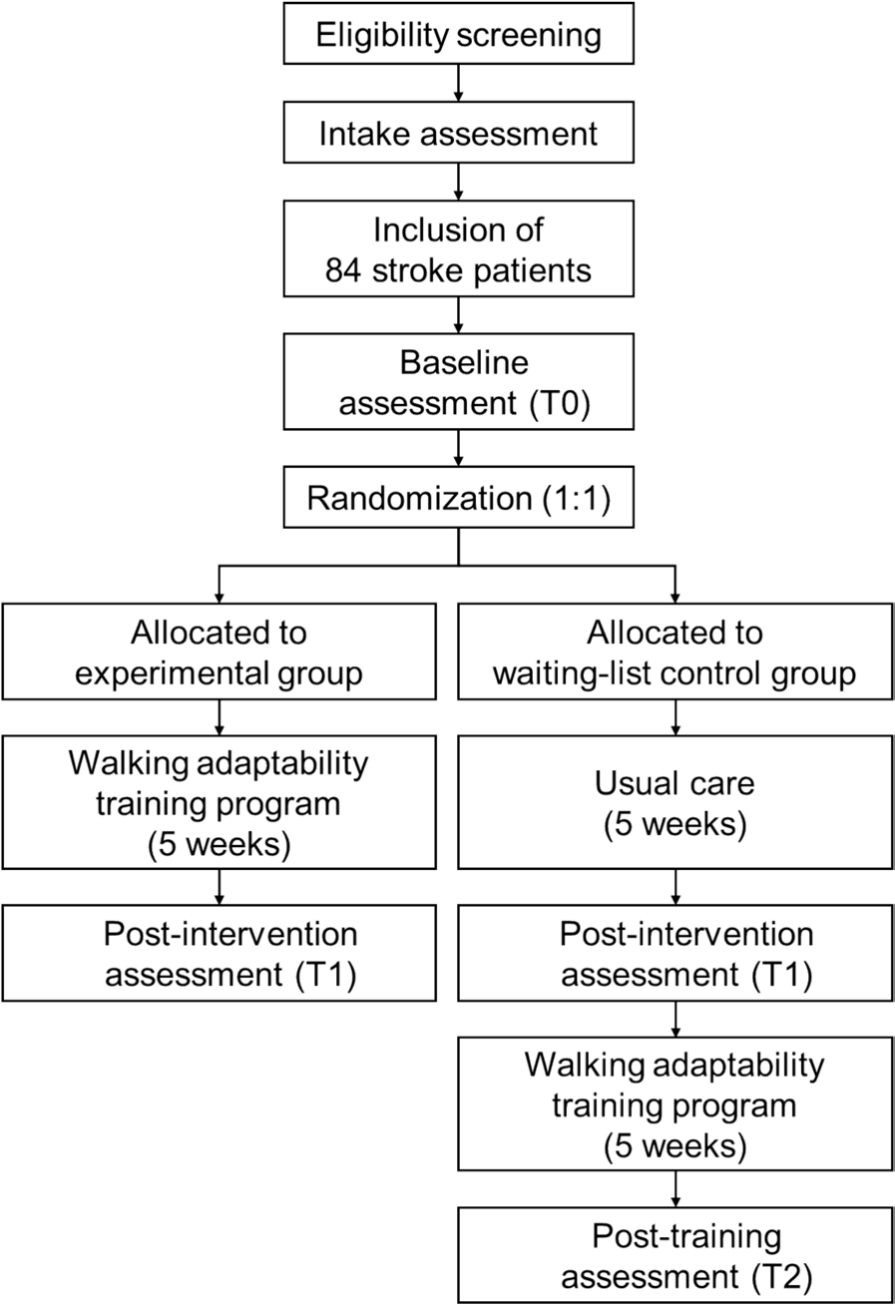
Timeline for the study

The timeline for this study is shown in Fig. 1. First, potential participants will be contacted by phone for eligibility screening and for collecting demographic characteristics. After provisional inclusion, participants are invited to the Gait Expertise Center at the Sint Maartenskliniek for an intake visit. During this visit, the inclusion criteria are further verified and definitive written informed consent is obtained. Following inclusion, several clinical tests are conducted for the characterization of residual stroke-related deficits. Approximately 2 weeks later, participants will undergo baseline assessment (T0), comprising a number of clinical and lab-based (using the GRAIL) tests at the Gait Expertise Center for obtaining the primary and secondary outcome measures, and 1 week of home-based monitoring of walking activity.

After conducting all the assessments during T0, participants are randomized to either the experimental group or the waiting-list control group. Participants will then either train or wait for 5 weeks, followed by a second assessment (T1) within 1 week after this period. Assessments at T1 are equal to baseline assessments (T0) in combination with the home-based monitoring period. Following T1, the waiting-list control group will receive the same walking adaptability training as the experimental group. They will undergo an additional assessment (T2) after the training period, identical to T0 and T1 assessments, to be used for responder analysis. Participants will also fill in a short questionnaire every month from baseline onward to report falls in the preceding month.

### Interventions

#### Experimental group

**Fig. 2:**
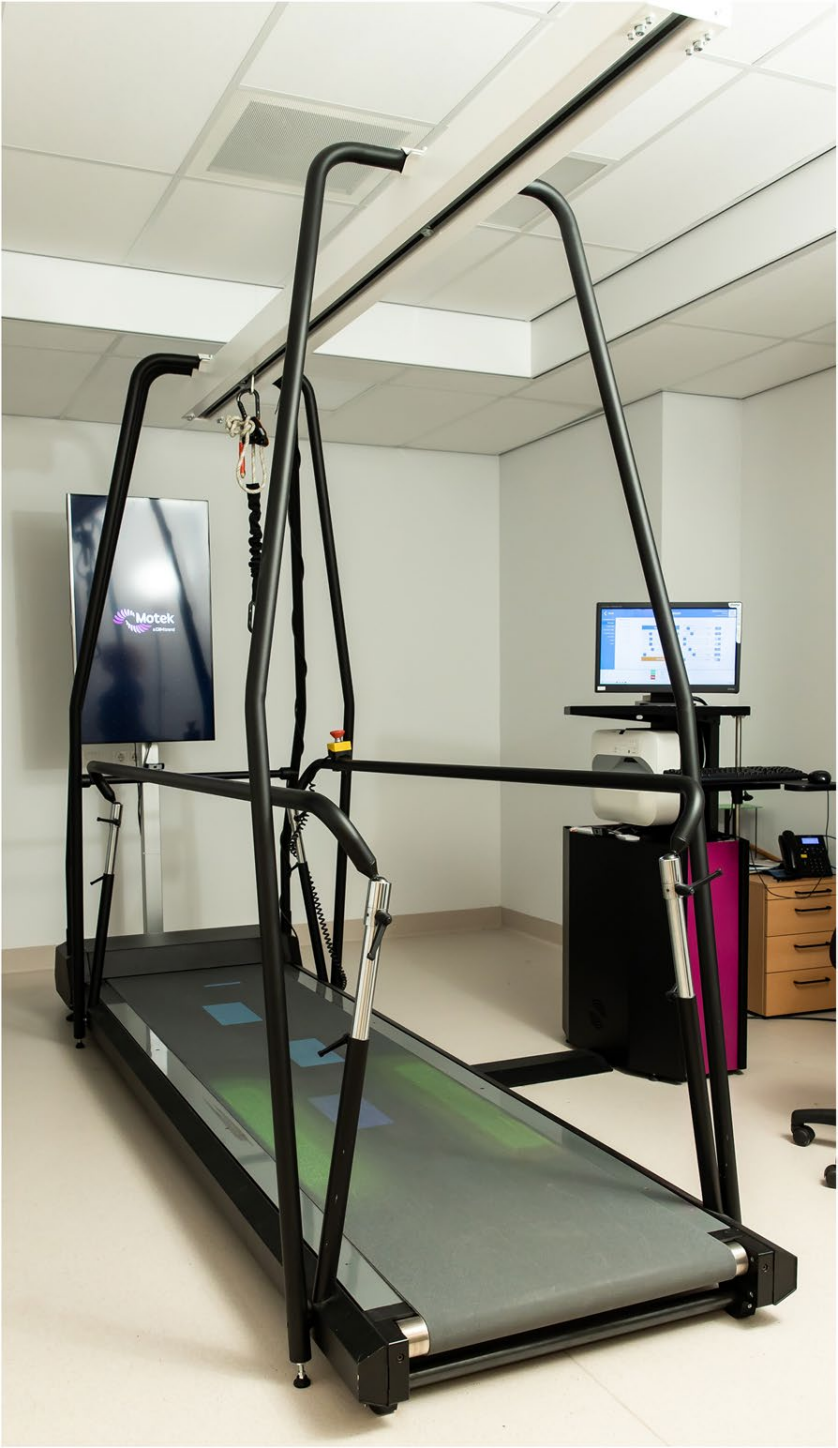
C-mill

The experimental group will receive a training program targeting walking adaptability. This training will be conducted on an instrumented treadmill with augmented reality (C-mill (Fig. 2), Motek Medical BV, the Netherlands). A projector next to the treadmill augments the treadmill surface with visual objects via projections (targets or obstacles) [8]. Embedded force plates in the treadmill enable online gait event detection, to be used for timing of the projections and to provide feedback on accuracy [8]. For safety purposes, participants wear a safety harness attached to a rail fixed to the frame of the C-mill.

The walking adaptability training program consists of ten 1-h sessions over a 5-week period. The training protocol is based on the previously published C-mill training protocol [20, 21], and further refined by our research team. Every training session will be guided by physical therapists with ample experience delivering C-mill training. Different physical therapists can guide the training sessions of one participant. Consistency of the content progression of the training between physical therapists is safeguarded by a protocol and logging of all training sessions.

Each training session consists of a set of exercises targeting the different components of walking adaptability: obstacle negotiation, target stepping, accelerating-decelerating, slalom walking and tandem walking. These components will be delivered separately, and in games combining multiple components. A total of eight different tasks (see Table 1) are included in the training protocol. Each task contains multiple levels of difficulty to enable progression between the training sessions (see Table 1). Progression of the training program is standardized for each task using success scores provided by the C-mill (see Appendix 1). The difficulty of the tasks can be varied through changes in walking (i.e., belt) speed and the addition of cognitive secondary tasks (i.e., dual tasking). The physical therapists are instructed to increase or decrease the difficulty level, based on the thresholds for the success scores defined by Timmermans et al. (2018) (see Table 2). If the proposed changes in difficulty are considered too challenging or too easy, the physical therapist is allowed to deviate, relying on their own clinical judgment. Training progression (including deviations) is logged by the therapist.

**Table 1:**
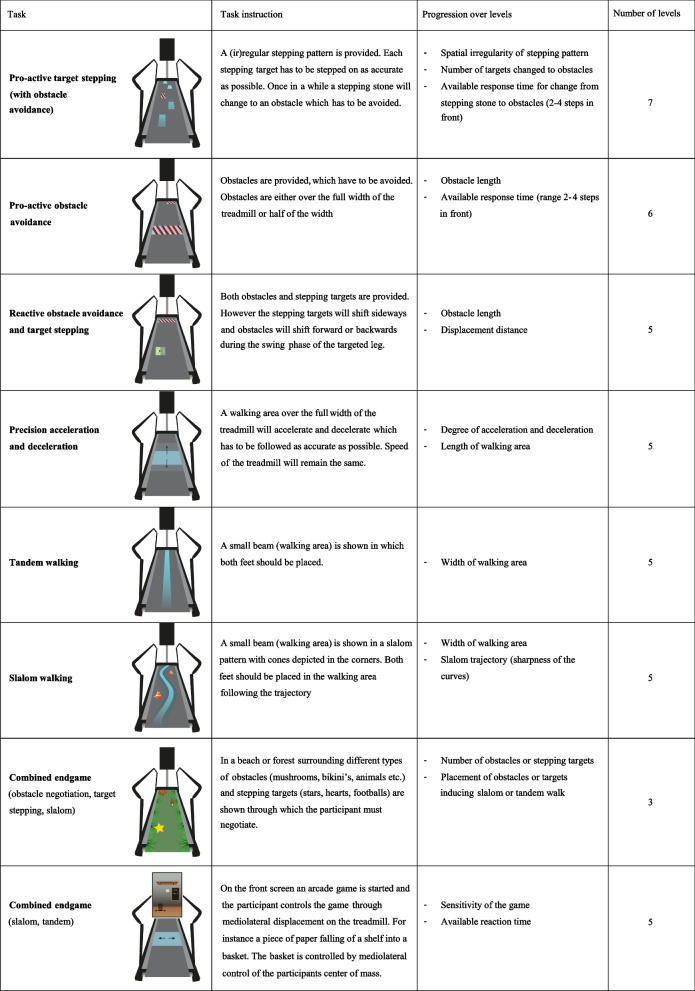
Descriptions of the 8 tasks included in the walking adaptability training program

**Table 2:** Thresholds used for standardized progression defined by Timmermans et al. (2019)

	Level down	Retain level	Level up
Pro-active target stepping, reactive target stepping and obstacle avoidance, precision acceleration and deceleration, tandem walking, slalom walking, combined endgames	Success score is 70% or lower	Success score is 70–90%	Success score is 90% or higher
Pro-active obstacle avoidance	Success score is 60% or lower	Success score is 60–80%	Success score is 80% or higher

The success score is the percentage of steps placed correctly relative to the projected targets or objects (see Appendix 1)

The first training session will be used to familiarize the participant with the training tasks and the C-mill, starting all tasks at the lowest level. The walking speed is set at the participant’s comfortable speed, as determined during T0. Walking speed is re-evaluated at session three and six. Since the progression of the training is based on the success score on each task, the training will be individualized from the second training session on to ensure a sufficiently challenging training program for the participant.

A break up to 1 week between two training sessions is allowed. Missed sessions due to a cancellation will be rescheduled as soon as possible. Adherence to the training sessions will be logged by the physical therapists. Adherence will be evaluated as the number of completed training sessions divided by 10 (total number of training sessions in the intervention).

During the training period, participants can continue their standard care. However, they are asked to stop physical therapy exercises focused on gait or balance training or other leg function during the training period. Furthermore, in case a participant regularly receives local intramuscular botulinum toxin injections (BTX) in the lower extremity, assessments are scheduled to minimize the influence of BTX on the measurements and no injections are scheduled during the walking adaptability training period. The protocol described in Van der Venis et al. (2021) is used to this end: T0 will be scheduled 4 weeks post-injections and T1 will be scheduled 10 weeks post-injections [20].

#### Waiting-list control group

During the 5-week waiting period, participants continue their usual care. The standard of care for people in the chronic phase after a stroke in the Netherlands can for instance be physical therapy or occupational therapy, dependent on the situation of the individual participant. No additional therapy or program is provided during the waiting-list control period. The number of therapy sessions during the waiting period will be recorded at T1. Following T1, participants receive the same walking adaptability training program as the intervention group. In case participants regularly receive BTX, assessments are scheduled to minimize the influence of BTX on the measurements and no injections are scheduled during the walking adaptability training period: T0 will be scheduled 4 weeks pre-injections, T1 will be scheduled 4 weeks post-injections, and T2 will be scheduled 10 weeks post-injections [20].

#### Criteria for discontinuing or modifying allocated interventions

Participants can leave the study at any time for any reason if they wish to do so, without consequence. The investigator can decide to withdraw a participant from the study for urgent medical reasons. In case a participant leaves the study before intervention allocation, the participant will be replaced, with a maximum of 10 replaced participants. Participants who leave the study after intervention allocation will not be replaced.

The intervention can be discontinued or modified due to the appearance of (serious) adverse events of a nature, severity and duration previously unknown, or of known (serious) adverse events occurring with an unexpectedly high frequency. However, given the absence of serious adverse events observed in the clinical practice with this type of training in this population, this is not expected.

### Participant retention

To promote retention in the study, all assessments and training sessions are scheduled in consultation with the participant. Throughout the study period, participants remain in direct contact with the administrative research assistant or the research coordinator (MN) to prevent missed appointments and reschedule appointments when necessary. To minimize drop out during the walking adaptability training program due to overexertion of the participant, the training intensity may temporarily be adjusted. A post-intervention assessment will always be encouraged to be completed for participants withdrawn from the study, to allow for intention-to-treat analysis.

### Assessments

All assessments are conducted by the research coordinator (MN), who is trained to perform all clinical tests and to operate the GRAIL. Participants are asked to wear comfortable clothing and shoes. During all assessments the use of orthopedic footwear and/or orthotic devices is allowed, except during the miniBESTest as per test instructions. No other walking aids are allowed. Table 3 shows the assessments at different timepoints.

**Table 3:**
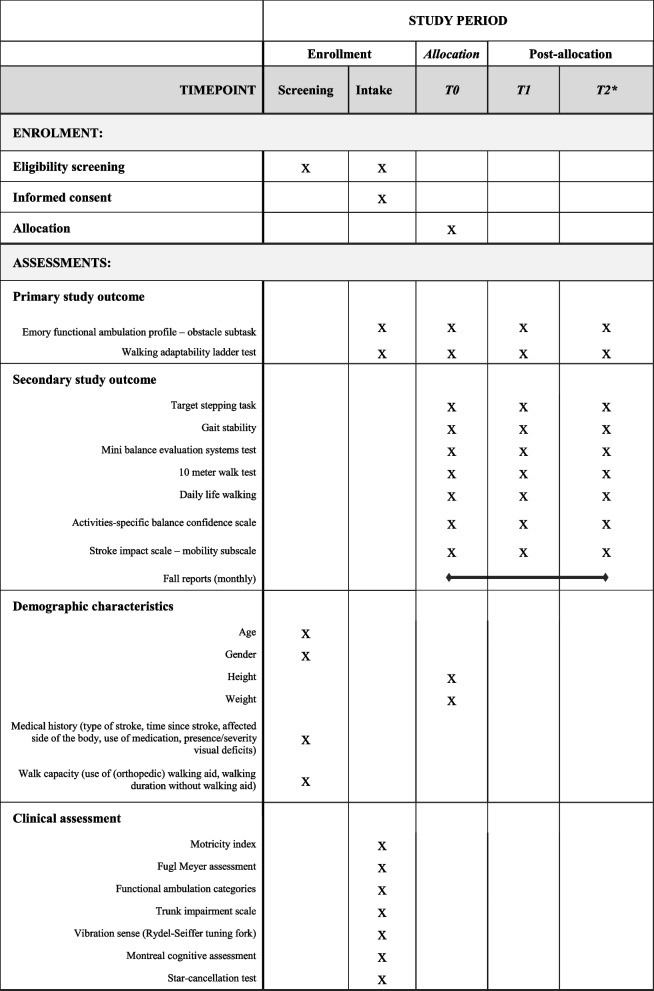
Overview of enrollment, interventions and assessments during study period: Recommendations for interventional trials (SPIRIT) figure

*Only for participants in waiting-list control group

### Demographics and clinical assessment

Demographic characteristics include age, sex, height and weight, type of stroke, time since stroke, affected side of the body, use of walking aid or orthopedic shoes or devices during walking, walking duration without walking aid, regular use of medication and presence and severity of visual deficits.

Clinical assessments consist of seven commonly used clinical tests: the motricity index (MI) for the lower extremity strength [19], the Fugl Meyer assessment (FMA) for the lower extremity level of motor impairment [22], the functional ambulation categories (FAC) for the level of independent walking [23], and the trunk impairment scale (TIS) for the level of motor impairment of the trunk [24]. Apart from the MI, these tests are recommended from the third Stroke Recovery and Rehabilitation Roundtable as a core set of the assessment of post-stroke lower extremity motor function, balance and mobility [25]. Vibration sensitivity is evaluated using the Rydel-Seiffer tuning fork on both feet at the lateral malleolus and the head of the first metatarsal bone [26]. The Montreal cognitive assessment (MoCA) is used to assess the level of cognitive function, and the star-cancellation test is used for visual hemispatial inattention [27, 28].

### Outcomes

For all outcomes, the primary endpoint of this trial is the measurement at T1. The reported statistics will include mean group differences and the associated 95% confidence intervals, as well as group means with SD.

#### Primary outcomes

We include two primary outcome measures for evaluating walking adaptability, the obstacle avoidance subtask of the Emory functional ambulation profile (EFAP-obstacle) and the walking adaptability ladder test (WALT) [29, 30]. The EFAP-obstacle has previously been used as an outcome measure for walking adaptability in a stroke population [8], but is expected to show a ceiling effect in our research population of community ambulators [31]. Therefore, we additionally used the newly developed WALT, which has been shown to be responsive to walking adaptability training effects in adults with hereditary spastic paraplegia and in children with developmental disorders [32, 33], but has not yet been validated in PwS.

During the EFAP-obstacle, participants traverse a standardized 5 m obstacle course at a comfortable walking speed, including stepping over two boxes (10(l) × 100(w) × 5(h) cm) put on the floor, and turning around a chair without touching it. Time to complete the EFAP-obstacle task is the main outcome parameter.

The WALT consists of a 10 m agility ladder with 18 stepping targets, which progressively decrease in length (2 cm per target, ranging from 69 to 35 cm). Participants are instructed to walk back and forth over the ladder as fast and accurately as possible, in single and double run conditions. During the single run, one foot is placed in each target. During the double run, both feet are consecutively placed in each target. Accuracy is rated by counting the number of stepping errors (wrong number of feet per target, touching the borders of the target or jumping in double run). Each error results in a 1-s time penalty. Execution of the WALT is recorded by video, following the recommendation of Kuijpers et al. (2020) to ensure reliability [30]. The video recordings are used to identify errors, and to record the time needed to complete the WALT. The main outcome measure is the sum of the time needed to complete the WALT plus the time penalty.

#### Secondary outcomes

##### Target stepping task

The target stepping task is performed on the GRAIL. Participants are instructed to step onto stepping stones projected on the treadmill belt, as accurately as possible. During 30 s of regular walking at a comfortable walking speed, step length and step width are assessed to reflect the comfortable walking pattern. Based on this pattern, an irregular stepping pattern is created. Changes in step length vary across -20%, -10%, 0%, 10%, 20% of the participants’ comfortable step length. Step width varies between -10 cm, 0 cm, 10 cm of the participant’s comfortable step width [34]. The optical motion analysis system (VICON, Oxford, United Kingdom) will be used to determine the accuracy of the placement of the participant’s foot for the first 150 steps. The accuracy of foot placement is quantified as the absolute Euclidean mean distance (mm) and its standard deviation (variable error) between the center of the foot and the center of the stepping stone [34].

##### Gait stability

Gait stability will be evaluated on the GRAIL. Perturbations are induced by lateral translation of the treadmill while participants walk at a comfortable walking speed. Inward (e.g., translation to the right at left heel strike) and outward (e.g., translation to the left at left heel strike) perturbations are administered in a semi-random order. The margin of stability and the center of mass excursion derived from 3D optical motion capture will be used as measures of gait stability [35].

##### Mini balance evaluation systems test (MiniBESTest)

The 14-item miniBESTest assesses anticipatory and reactive postural control, sensory orientation and dynamic gait [36]. The separate items will be scored on a 3-point ordinal rating scale (0–2 points), with higher scores indicating better performance. The outcome measure is the summed score over all items. Participants will perform the test barefoot. This test has been found to be a valid, reliable tool for evaluating balance in PwS [36, 37].

##### 10-m walk test (10MWT)

During the 10MWT, participants walk 13 m in a straight line of which the last 10 m are timed using a stopwatch. The first 3 m are used to accelerate to the comfortable walking speed. When the first foot crosses the 3 m line the timer starts. When the first foot crosses the 13 m line the timer stops and participants are instructed to stop walking. Participants perform the test three times at a comfortable walking speed. The outcome measure is the mean time of these three performances [38].

##### Daily life walking

Daily life walking will be evaluated in terms of the amount of walking, and in terms of spatiotemporal gait parameters (e.g., stride time). For the amount of walking, participants will wear an Activ8 Physical Activity Monitor (Activ8, Remedy Distribution Ltd., Valkenswaard, The Netherlands) attached to one of the upper legs for seven consecutive days. Using customized algorithms the time spent in different activity classes (lying, sitting, standing, walking, running, cycling) will be determined based on the acceleration data collected by the activity monitor [39]. The outcome measure for this study is the mean time spent walking per day averaged over the total number of days the monitor is worn. For spatiotemporal gait parameters, participants will wear three inertial measurement units for 3 to 4 days: one on each foot and one on the lower back. Customized algorithms will be used to identify gait bouts, and derive the spatiotemporal parameters from these gait bouts. During these monitoring periods, participants are allowed to use all types of (orthopedic) walking aids, as they would normally use in daily life.

##### Activities-specific Balance Confidence scale (ABC scale)

The ABC scale is a questionnaire consisting of 16 items asking about how confident participants are to maintain their balance while performing different activities of daily life. Scores range from 0 to 100% confident [40].

##### Stroke Impact Scale–Mobility Subscale (SIS-mobility)

The SIS-mobility is a questionnaire evaluating disability and health-related quality of life after a stroke [41]. The Dutch Mobility subscale will be used in this study. Scores will be given on a 5-point scale, with higher scores indicating a lower impact of the stroke on daily life.

##### Fall reports

Each month, the participants are asked how many times they have experienced a fall in the preceding month through an online survey (CastorEDC) sent by email to the participants. A fall is defined as “an event which results in a person coming to rest inadvertently on the ground or other level” as defined by the World Health Organisation [42]. If one or two falls are reported, the participant is asked to report a short description of these events and the circumstances. In case the participant reports more than two falls, additional information is asked about the last two events.

### Data collection, management, and monitoring

Each participant receives a unique unidentifiable code generated by Castor EDC (www.castoredc.com). This code is assigned to the participant in order of inclusion of the subject. The identification code list is a protected file and is held separate from the data. This file is only accessible for researchers and administrative assistants working on the project. The collected data will be entered into Castor EDC. Handling of data will comply with the Dutch Personal Data Protection Act. After completion of the study, data will be stored for 15 years according to the Dutch national legislation. Researchers affiliated with the Sint Maartenskliniek and this study will have access to the data.

Monitoring and quality assurance will be established according to the NFU (Dutch Federation of University Medical Centres) guidelines for negligible risk intensity. Monitoring will be executed by an independent qualified monitor.

### Safety

The classification for risk is estimated at negligible risk. In case of an adverse event that is reported spontaneously by the subject or observed by the investigator or his staff, this will be recorded and reported to the accredited METC. All adverse events will be followed until they have abated, or until a stable situation has been reached. Depending on the event, follow-up may require additional tests or medical procedures as indicated, and/or referral to the general physician or a medical specialist.

### Sample size

The sample size calculation was based on previous work comparing pre- to post-walking adaptability training intervention in a stroke population using the EFAP-obstacle (effect size: 0.7) [8]. Assuming a change of 10% of the intervention effect size in the waiting list control group, each group should include 47 participants to achieve a power of 80%, setting the alpha level at 0.025 to correct for two primary outcome measures. A correction factor was applied to account for a correlation (*r* = 0.5) between T0 and T1 [43], resulting in 35 participants per group. Taking 15% attrition into account, in total 84 participants will be randomized.

### Statistical methods

To evaluate the effect of the training program, an intention-to-treat analysis will be conducted as the primary analysis. All participants who are randomized to one of the two study arms (including those who drop out during the intervention period, or never started the intervention) will be included in the primary analysis. In addition, a per-protocol analysis will be performed, excluding participants in the experimental group not completing the training program or participants in the waiting-list control group receiving training targeting walking adaptability. The training program is considered complete if at least 8 out of 10 training sessions are concluded.

The effect of the walking adaptability training on primary and secondary outcomes will be tested with a linear mixed model. The scores at post-intervention (T1) will be used as the dependent variable and group allocation (intervention or waiting-list control) as the independent variable; covariates are baseline scores of the dependent variable (T0) and the stratification variable (MI score), and participant ID as a random factor. A Bonferroni correction will be applied due to the two primary outcome measures; therefore significant effects are assumed for *p* < 0.025. Effects will be presented as the mean difference with 95% confidence intervals.

For the responder analysis, we will use the data of all participants who completed the training program in the study, because all participants underwent the walking adaptability training either as the experimental group or directly following the waiting-list period. To identify baseline participant characteristics that predict a response to training (defined as scores on the EFAP-obstacle or WALT), a linear regression model will be used. Predictors for response to the training will be collected during the intake assessment and T0 for both groups. To determine response to training, the relative change in EFAP-obstacle and WALT at T1 for the experimental group and T2 for the waiting-list control group will be used. Age, Motricity Index score and the miniBESTest score will be tested for their predictive value, and an exploratory analysis will be performed on other variables that may predict outcome.

## Discussion

This two-armed, superiority, randomized, waiting-list controlled trial will evaluate the efficacy of a walking adaptability training program on a treadmill with augmented reality to improve walking adaptability in people in the chronic phase after stroke. Based on previously reported beneficial effects of a walking adaptability training program [10, 11, 13], it is hypothesized that walking adaptability will improve from T0 to T1 in the intervention group receiving a walking adaptability training program, while it remains unchanged for the waiting-list control group receiving usual care. This study aims to provide high-grade evidence for the efficacy of a walking adaptability training program for PwS.

To evaluate the efficacy of a walking adaptability training, a primary outcome measure assessing walking adaptability is key. However, no gold standard test for the assessment of walking adaptability exists. We opted for two main outcome measures to evaluate walking adaptability: the EFAP-obstacle and the WALT. The EFAP-obstacle has been used in a previous training study targeting walking adaptability [8, 20], and could thus be used to calculate an adequate sample size. However, it is expected that the EFAP-obstacle has a ceiling effect in people with a high level of functioning, due to the use of small obstacles and lack of time pressure [4]. Because participants in this study likely have a high walking function, we cannot rule out that the EFAP-obstacle lacks sensitivity for detecting a change in walking adaptability due to the training. In contrast to the EFAP-obstacle, the WALT is expected to be more sensitive. This test was originally developed for evaluating walking adaptability in children and has shown good to excellent reliability and good construct validity for walking adaptability [30]. No apparent ceiling effects have been observed in adults with hereditary spastic paraplegia or in children with developmental disorders [32, 33]. Furthermore, pilot tests with PwS showed that even those with good motor recovery found the task challenging, as evidenced by higher (i.e., poorer) scores compared to yet unpublished data from healthy controls in the same age range. Moreover, previous studies from our group demonstrated that the WALT was responsive to the effects of walking adaptability training in children with developmental coordination disorders and in adults with hereditary spastic paraplegia [32, 33]. Based on these promising findings, we decided to also include the WALT as a primary outcome measure.

This study is the next step to determine the efficacy of a task-specific walking adaptability training program using augmented reality to improve walking adaptability in PwS. An improved walking adaptability is beneficial for community ambulation. In case the results confirm the efficacy of this walking adaptability training, it could reduce the persistent high fall risk associated with impaired walking adaptability [44]. Subsequently, the responder analysis is expected to provide insights into which individuals are most likely to benefit from this type of training.

## Data Availability

Public access will be granted for primary and secondary outcomes, predictive values (age, Motricity index), post hoc motion capture analysis and statistical code. No personal information or medical history that leads to the participants’ identity will be accessible.

## Appendix 1

### Details on the success score used for standardized progression

The C-mill is an instrumented treadmill with embedded force plates in the treadmill [8]. These force plates enable online gait event detection using the trajectories of the center of pressure [45]. With this online gait event detection a performance score per foot per task can be determined during the execution of that particular task. This performance score is the percentage of steps placed correctly relative to the projected targets or objects. Thus, within the assigned walking areas for tandem walk, slalom walk and acceleration-decelerations, on the projected stepping targets, and outside the obstacles. Dependent on the task the mean of the provided performance scores will be calculated afterward by the physical therapist as a success score (see Table 4). These success scores determine if the level of the task should be decreased by one level, should be retained or should be increased by one level in the next training session. Since all tasks will be evaluated separately, the change in level can deviate over the tasks as the training program progresses.

**Table 4:** Performance scores provided by C-mill and success scores used for progression

Task	Provided performance scores	Success score used for progression
**Pro-active target stepping (with obstacle avoidance)**	- Percentage of targets hit with left leg (TL) - Percentage of targets hit with right leg (TR) - Percentage of obstacles avoided with left leg (OL) - Percentage of obstacles avoided with right leg (OR)	Mean percentage of all targets hit and obstacles avoided: (TL + TR + OL + OR)/4
**Pro-active obstacle avoidance**	- Percentage of obstacles avoided	Percentage of obstacles avoided
**Reactive obstacle avoidance and target stepping**	- Percentage of targets hit (T) - Percentage of obstacles avoided (O)	Mean percentage of all targets hit and obstacles avoided: (T + O)/2
**Precision acceleration and deceleration**	- Percentage of steps within area with left leg (stepsL) - Percentage of steps within area with right leg (stepsR)	Mean percentage of all targets hit and obstacles avoided: (stepsL + stepsR)/2
**Tandem walking**	- Total score of steps placed on the projected beam (with 100 as maximal score)	Total score of steps placed on the projected beam
**Slalom walking**	- Percentage of steps within area with left leg (stepsL) - Percentage of steps within area with right leg (stepsR)	Mean percentage of all targets hit and obstacles avoided: (stepsL + stepsR)/2
**Combined endgame 1** (obstacle negotiation, target stepping, slalom)	- Percentage of targets hit (T) - Percentage of obstacles avoided (O)	Mean percentage of all targets hit and obstacles avoided: (T + O)/2
**Combined endgame 2** (slalom, tandem, cognitive dualtask)	- Total score, dependent on the game	Mean percentage of combined endgame 1, since total score depends on the game

## Abbreviations

10MWT: 10-Meter walk test
ABC Scale: Activity-specific balance confidence scale
BTX: Botulinum toxin
CWZ: Canisius Wilhelmina Ziekenhuis
EFAP: Emory functional ambulation profile
FAC: Functional ambulation categories
FMA: Fugl-Meyer assessment
GRAIL: Gait real-time analysis interactive lab
MI: Motricity index
MiniBESTest: Mini balance evaluation system test
MoCA: Montreal cognitive assessment
PwS: People with stroke
SIS: Stroke impact scale
TIS: Trunk impairment scale
WALT: Walking adaptability ladder test
